# Alternation of β-catenin and CD44s Immunoexpression in Different Histopathological Grades of Oral Squamous Cell Carcinoma

**DOI:** 10.31557/APJCP.2020.21.5.1181

**Published:** 2020-05

**Authors:** Massoumeh Zargaran

**Affiliations:** *Department of Oral and Maxillofacial Pathology, Faculty of Dentistry, Kurdistan University of Medical Sciences, Sanandaj, Iran. *

**Keywords:** Oral squamous cell carcinoma, β-catenin, CD44s, immunohistochemistry

## Abstract

**Objective:**

Cell-cell adhesion molecules play an essential role in cell growth and differentiation. β-catenin and CD44s are two adhesion molecules which their expression changes are correlated to loss of differentiation and gain of an invasive epithelial phenotype. Oral squamous cell carcinoma (OSCC) is the most common malignancy of oral cavity. Aim of this study was to compare β-catenin and CD44s expression in different histopathological grades of OSCC.

**Methods:**

β-catenin and CD44s expression were evaluated in 10 well differentiated OSCC (group A) and 10 moderately/poorly differentiated OSCC (group B) using immunohistochemistry.

**Results:**

β-catenin membranous and nuclear/cytoplasmic expression were significantly different between groups A and B. CD44s membranous expression was insignificant amongst the two groups.

**Conclusion:**

Expression of β-catenin and CD44s alter in different histopathological grades of OSCC. It seems that more rate of aberrant cytoplasmic and/nuclear expression and less membranous expression of β-catenin can lead to significantly lower degree of cell differentiation in OSCC.

## Introduction

Cell-cell adhesion molecules play a fundamental role in cell growth and differentiation (Laxmidevi et al., 2010). These molecules mediate the adhesion of epithelial cells to each other and preserve integrity of tissue structure (Lopes et al., 2009; Rosado et al., 2013). 

Oral squamous cell carcinoma (OSCC) is the most common malignancy of oral cavity accounting for over 90% of all malignancies in this area (Hema et al., 2014). Invasion and metastasis are among the characteristics of OSCC. Cell escape from tumor is the first step for invasion and metastasis. This event occurs as a result of changes in expression of adhesion molecules and disruption of cell-cell adhesion (Cai et al., 2008). 

β-catenin and CD44 are two adhesion molecules (Laxmidevi et al., 2010; Krump and Ehrmann, 2013). β-catenin is a multifunctional 92-kDa protein (Laxmidevi et al., 2010) which involves in two completely different processes namely intercellular adhesion and signal transduction (Cai et al., 2008). CD44 is a cell surface glycoprotein and the main receptor for hyaluronan (Krump and Ehrmann, 2013; Hema et al., 2014). Standard isoform of CD44 (CD44s) is the smallest and the most abundant isoform of this protein (Louderbough and Schroeder, 2011). Evidently, expression of β-catenin and CD44s changes are correlated to loss of differentiation and gain of an invasive epithelial phenotype (Simionescu et al., 2008; Hema et al., 2014; Zaid, 2014). This study aimed to compare expression of β-catenin and CD44s in different histopathological grades of OSCC. 

## Materials and Methods

*Patients*

OSCC samples belong to a previous study (Zargaran et al., 2018) were used in current research. They were microscopically evaluated and classified according to histopathological differentiations including 10 cases of well differentiated (WDOSCC; group A) and 10 cases of moderately/poorly differentiated OSCC (MD/PDOSCC; group B). Ethical approval of the current study was obtained from the Ethical Committee for Research of Hamadan University of Medical Sciences.

*Immnuohistochemistry*

Imunohistochemical study was performed by Envision detection system, using β-Catenin (monoclonal mouse anti-human Beta-Catenin; Clone β-Catenin-1, Ready-to-Use, Dako, Denmark) and CD44s (monoclonal mouse anti-human phagocytic glycoprotein-I; Clone DF 1485 Code-Nr. M 7082, Dako, Denmark) antibodies. Briefly, 4-μm tissue sections were cut from paraffin blocks, mounted on poly-L-lysine-coated slides, deparaffinized and rehydrated. Endogenous peroxidase activity was blocked using 3% hydrogen peroxide for 5 minutes. For antigen retrieval slides were boiled in citrate buffer (pH=6 for CD44, High pH for β-catenin) inside an autoclave for 30 minutes. Then, the slides were incubated with antibodies for 60 minutes washed in phosphate buffered saline (PBS) for 10 minutes whereas an Envision (Code: K5007; HRP, Dako, Denmark) was used for 30 minutes. Subsequently, the sections were washed with PBS for 10 minutes and DAB was used as chromogen. Finally, the slides were counterstained with Harris hematoxylin, dehydrated, and mounted.

For β-catenin, membranous /cytoplasmic /nuclear staining and for CD44, membranous staining was considered as positive immunoreactions. Immunoreactivity for β-catenin and CD44 was assessed using IHC immunoreactivity score. Percentage of positive membranous cells were scored: 0 (<20% stained cells), 1 (21-40% ), 2 (41-60%), 3 (61-80%), and 4 (>80%) and staining intensities were graded: 0=no expression, 1=weak, 2=moderate and 3=strong. Subsequently, IHCmembranous score was calculated by positive membranous stained cell score × staining intensity score. Also, according to this method β-catenin IHCcytoplasmic and/ nuclear score was determined in the studied groups. 

*Statistical Analysis*

Data were statistically analyzed using SPSS V16.0 and Independent T test while P<0.05 was considered as significant level.

## Results

There were 5 males and 5 females in each studied groups of A and B whereas mean age were 59 (range 35-78 years) and 60 (38-82 years), respectively. The sites of cases for group A were buccal mucosa (n=3), alveolar ridge (n=5), labial vestibule(n=1) and tongue (n=1) while for group B were tongue (n=6), floor of the mouth (n=2), gingiva (n=1) and retromolar pad (n=1). 

**Figure 1:**
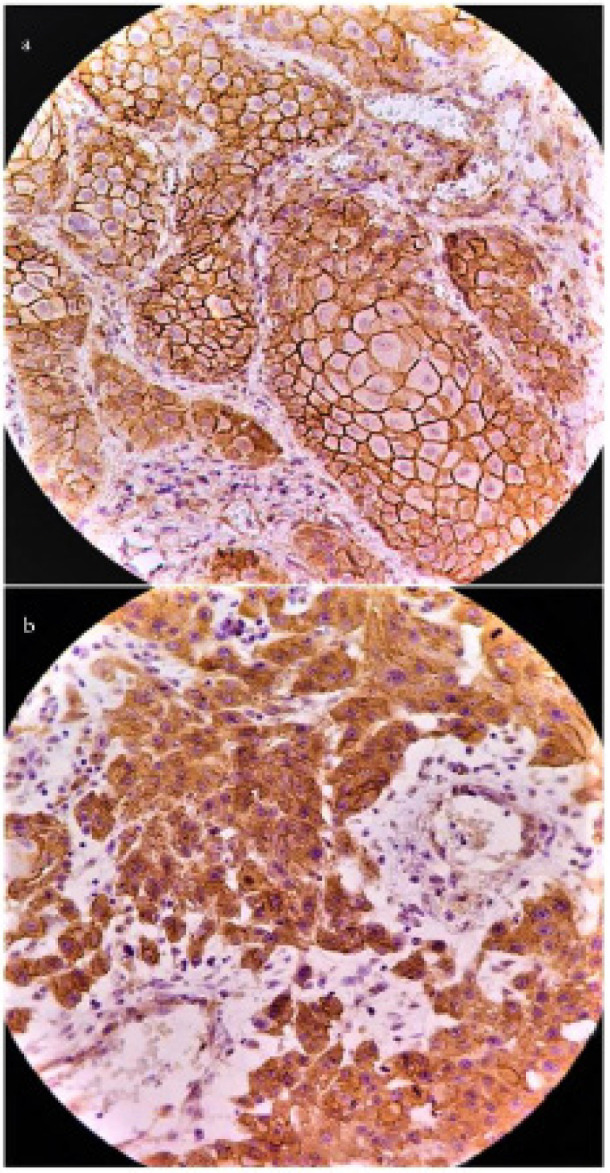
β-catenin Immunostaining in Studied Groups; a, well differentiated OSCC; b, moderately/poorly differentiated OSCC (a and b, 400× magnification).

**Figure 2:**
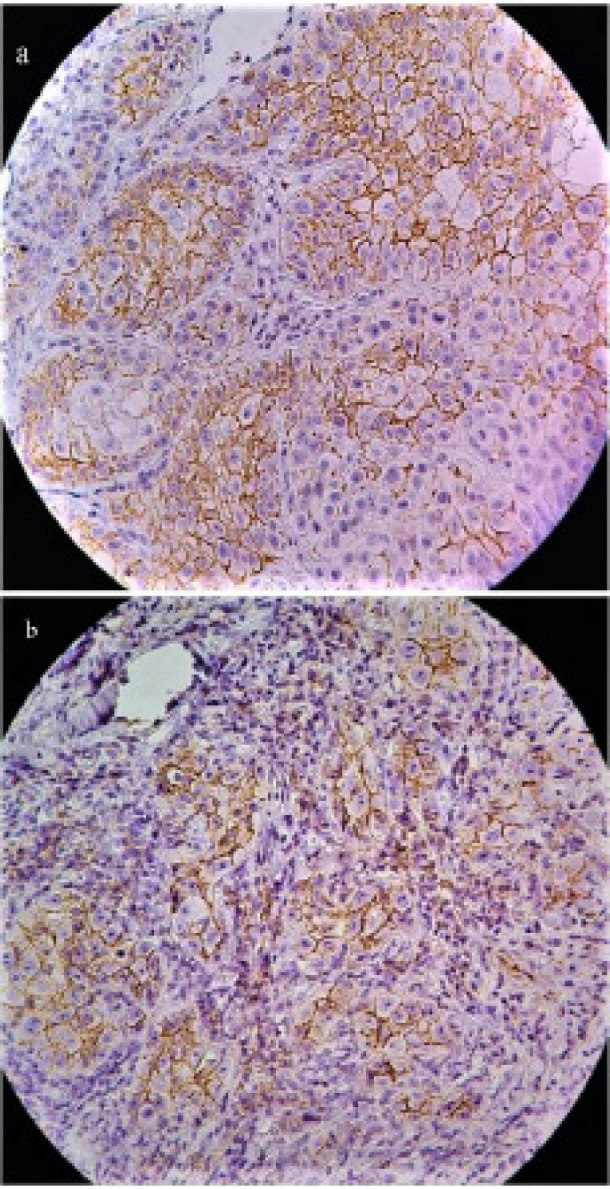
CD44 Immunostaining in Studied Groups; a, well differentiated OSCC; b, moderately/poorly differentiated OSCC (a and b, 400× magnification)

Kolmogorov-Smirnov test confirmed normal distribution of β-catenin and CD44s IHCmembranous score as well as β-catenin IHCcytoplasmic and/ nuclear score. Except for more superficial epithelial cell layers, other layers (especially basal and suprabasal cell layers) in normal tissue adjacent to some of OSCC expressed positive membranous staining of β-catenin (and CD44s). However, no evidence of expression in cell cytoplasm and nuclei was observed in them. β-catenin membranous, cytoplasmic and nuclear immunostaining (Figure 1) were observed in 100% of OSCC cases. Similarly, CD44s membranous immunostaining (Figure 2) was detected in all of the studied cases.

Mean of β-catenin IHCmembranous was 3.400±2.366 in group A and 0.600±0.843 in group B while 2.300±0.483 and 3.800±2.043 were mean of β-catenin IHCcytoplasmic and/ nuclear in group A and B, respectively. β-catenin, IHCmembranous score and IHCcytoplasmic and/ nuclear score were significant differences between group A and B (P=0.002 and P=0.047, respectively). Mean CD44s IHCmembranous score was 4.700±2.359 in group A and 3.000±1.825 in group B. CD44s IHC scoremembranous was insignificant between the groups (P=0.088).

## Discussion

Whole of the samples were positive for β-catenin immunostaining and showed the three types of membranous, cytoplasmic and nuclear staining. In previous studies by Zaid (2014) and Rosado et al., (2013) the percentage of stained samples were smaller than of the current study. Similar to the findings by Laxmidevi et al., (2010), Zaid (2014) and Ravindran et al., (2015) a significant difference was noted in β-catenin membranous expression for different grades of OSCC in this study. Oppositely, Liu et al., (2010), Rosado et al., (2013) and Balasundaram et al., (2014) found no significant difference in expression of β-catenin for different grades of OSCC. In present study, β-catenin cytoplasmic and/nuclear immunostaining were significantly different amongst groups A and B in agreement to the findings by Zaid (2014) but opposite to those of Zhang et al., (2014). 

Lopes et al., (2009) reported a significant association between cytoplasmic expression of β-catenin and histopathological grade but no significant association was noted with its nuclear expression.

Controversy in results of studies regarding expression β-catenin (and also other marker of this study/CD44s) can be due to a number of factors such as heterogeneity of tissue samples studied, difference in sample size, immunohistochemical staining/antigen retrieval techniques and scoring criteria. However, reduction in β-catenin membranous staining and a shift towards cytoplasmic (and/or nuclear) immunostaining by an increase in OSCC grade is a significant finding of the current study and also among the most of the previous studies (Laxmidevi et al., 2010; Liu et al, 2010; Rosado et al., 2013; Zaid, 2014; Angadi et al., 2016). 

β-catenin is normally located and expressed on the cell membrane of normal cells (Zaid, 2014). In this region, β-catenin plays a role in E-cadherin-mediated cell adhesion (Cai et al., 2008). It binds to the cytoplasmic domain of E-cadherin which forms E-cadherin/β-catenin complex (Zaid, 2014) and establishing cell-cell contact (Rosado et al., 2013). The stability and integrity of this complex can be disrupted by the dissociation of β-catenin from E-cadherin. In this case, cell adhesion is lost and the cells become migratory and motile (Cai et al., 2008). According to the current findings: (I) absence of β-catenin membranous immunostaining in a number of neoplastic epithelial cells in groups A and B revealed that in OSCC changes such as cell cohesion loss and cellular motility occur irrespective of its degree of differentiation (II) significantly lower membranous immunostaining in MD/PDOSCC compared to WDOSCC may indicate further dissociation of cells from the primary tumor bulk and easier occurrence of invasion and even metastasis in this group of tumors. 

Dissociation of β-catenin from E-cadherin decreases its membranous expression and leads to accumulation of free β-catenin in cytoplasm and its subsequent move into the cell nucleus (Kaur et al., 2013; Rosado et al., 2013). Normally, free β-catenin in cytoplasm is phosphorylated via attachment to a complex namely degradation complex which is composed of Axin, APC and GSK3 proteins and is degraded by the ubiquitin-proteasome rapidly (Laxmidevi et al., 2010). Mutation in APC and Axin genes or β-catenin gene itself, dysfunction of GSK3 and/or Wnt signaling pathway activation can impede proper functioning of the degradation complex (Laxmidevi et al., 2010; Yun et al., 2010; Rosado et al., 2013). This can lead to accumulation of β-catenin in cytoplasm and promote its subsequent translocation into the nucleus. β-catenin serves as an oncogene in nucleus (Yun et al., 2010). Binding of β-catenin to members of TCF/LEF family leads to activation of a number of proto-oncogenes such as c-Myc and cyclin D1 (Lopes et al., 2009).

In this study, β-catenin cytoplasmic/nuclear expression was noted in both groups of WOSCC and MD/PDOSCC. Apparently, aberrant activity and expression of β-catenin in OSCC is not exclusive to a certain histopathological grade. Also, significantly higher frequency of this form of expression in group B can be attributed to the occurrence of higher changes and abnormalities in complex intracellular mechanisms of MD/PDOSCC compared to WDOSCC. MD/PDOSCC cases probably suffer from higher abnormalities such as mutation of APC, Axin and/or β-catenin genes, dysfunction of GSK3 or Wnt signaling pathway activation compared to WDOSCC. Significantly higher β-catenin cytoplasmic and/nuclear expression in group B compared to group A suggest that OSCCs with lower differentiation grade possess greater accumulation of genetic defects. These genetic defects lead to loss of epithelial phenotype and migratory cell control as an indication of aggressive behavior of tumor (Chaw et al., 2012). 

Whole of the samples showed positive staining for CD44s marker. Simionescu et al., (2008) reported one and five cases of moderately and poorly differentiated OSCC with negative CD44s immunostaining, respectively. Similar to Krump and Ehrmann (2013); in the current study the difference in expression of CD44s was not significant between groups A and B. In contrast, Hema et al. (2014) demonstrated a significant difference in this expression between different grades of OSCC. Additionally, the most expression was noted in WDOSCC group in the present study similar to Simionescu et al. (2008) where higher frequency of cells with positive CD44s immunostaining in WDOSCC group was found compared to PDOSCC group. 

CD44s is a molecule with a diversity of functions which can play a role in both cell adhesion and cell motility/migration while it has extracellular, transmembrane and cytoplasmic domains. Function of CD44s highly depends on a ligand that binds to its extracellular domain. Hyaluronan is the most recognized ligand for CD44s. It is ubiquitous in extracellular and pericellular matrix and is synthesized as a high molecular weight (HMW) mass. CD44-HMW hyaluronan interaction causes the attachment of CD44 tail to Merlin (a tumor suppressor protein) which induces signals that mediate cell-cell and cell-extracellular matrix adhesion/ECM (Louderbough and Schroeder, 2011). However, tumoral cells release hyaluronidase which degrades HA and creates low molecular weight (LMW) hyaluronan fragments (Afify et al., 2008; Louderbough and Schroeder, 2011). Binding of LMW hyaluronan to CD44 leads to proteolytic cleavage of extracellular domain of CD44. In this process, ERM replaces Merlin and activates the signaling molecules that promote cytoskeleton remodeling and cellular migration (Louderbough and Schroeder, 2011). 

According to the previous studies; occurrence of cleavage in CD44s by LMW hyaluronan can down-regulate CD44s expression (Afify et al., 2008). CD44s immunostaining in some OSCC cells, not all of them, in groups A and B suggests that the cleavage of CD44s and induction of CD44 dependent cell migration irrespective to degree of differentiation in this malignancy. CD44s expression was not significantly different between MD/PDOSCC and WDOSCC groups. Although lower expression of CD44s in MD/PDOSCC may suggest greater loss of cell adhesion and easier tumoral cells detachment from each other and from ECM compared to WDOSCC. CD44 can activate or inhibit cell invasion and migration via interaction with LMW or HMW hyaluronan, respectively. In other words, CD44 mediated signaling pathways highly depend on extracellular conditions and changes in the microenvironment and can be significantly variable among different cell types or even among similar cells (Louderbough and Schroeder, 2011). Irrespective to a small sample size in this study, absence of a significant difference CD44s may indicate relatively similar microenvironment and ECM of the studied samples. 

In conclusion, expression of β-catenin and CD44s alter in OSCC irrespective of the related histopathological grade. It seems that more occurrence of abnormal changes in β-catenin, more rate of aberrant cytoplasmic and/nuclear expression alongside with less membranous expression can significantly lead to lower degree of cellular differentiation in OSCC. 
